# Extraction-Free Colorimetric RT-LAMP Detection of SARS-CoV-2 in Saliva

**DOI:** 10.3390/diagnostics13142344

**Published:** 2023-07-11

**Authors:** Ane Rivas-Macho, Ane Sorarrain, José M. Marimón, Felipe Goñi-de-Cerio, Garbiñe Olabarria

**Affiliations:** 1 Gaiker, GAIKER Technology Centre, Basque Research and Technology Alliance, Parque Tecnológico, Ed. 202, 48170 Zamudio, Spain; 2 Molecular Biology and Biomedicine PhD Program, University of the Basque Country UPV/EHU, 48940 Leioa, Spain; 3 Biodonostia Health Research Institute, Infectious Diseases Area, Microbiology Department, Osakidetza Basque Health Service, Donostialdea Integrated Health Organization, 20014 San Sebastián, Spain

**Keywords:** RT-LAMP, SARS-CoV-2, saliva, calcein, extraction-free

## Abstract

The pandemic situation caused by severe acute respiratory syndrome coronavirus 2 (SARS-CoV-2) has highlighted the need of fast, simple, and cost-effective tests for the diagnosis of emerging pathogens. RT-qPCR has been established as the reference technique for the diagnosis of SARS-CoV-2 infections. This method requires a time-consuming protocol for the extraction of the nucleic acids present in the sample. A colorimetric reverse transcription loop-mediated isothermal amplification using the calcein molecule combined with a simple extraction-free method for saliva samples (calcein RT-LAMP) has been developed. Samples are heated 95 °C for 10 min before amplification at 63 °C for 40 min. The results can be observed by fluorescence or by the naked eye with a color change from orange to green. The method was compared with commercialized available colorimetric and fluorescent RT-LAMP kits. The developed method shows better sensitivity and specificity than the colorimetric commercial RT-LAMP and the same as the fluorescent RT-LAMP, without the need of a fluorescent reader. Moreover, the calcein RT-LAMP has, compared to RT-qPCR, a sensitivity of 90% and a specificity of 100% for saliva samples with a Ct ≤ 34, without the need for expensive RT-qPCR instruments, demonstrating the potential of this method for population screening.

## 1. Introduction

The global outbreak caused by the severe acute respiratory syndrome coronavirus 2 (SARS-CoV-2) was declared a pandemic by the World Health Organization (WHO) on 11 March 2020. As of April 2023, this virus has caused more than 765 million confirmed cases all over the world since December 2019 [1,2]. Although the vaccination of the population has considerably improved the situation of the pandemic by decreasing mortality rates, it is still important to control the new infections. The existence of regions of the world with low population immunity, coupled with the fact that fewer tests are being carried out, means that fewer infections are detected, which could increase the risk of new local outbreaks of the disease and the appearance of new variants of concern that increase pressure on healthcare services and collapse hospitals.

The reference method for the detection of the virus SARS-CoV-2 recommended by the WHO is an RNA extraction from a nasopharyngeal and oropharyngeal swab followed by an RT-qPCR amplification [3]. RT-qPCR has demonstrated to be accurate, highly sensitive, and specific, but it is hardly scalable to perform outside the laboratory due to the need for sophisticated thermocycler equipment [4] and the laborious and time-consuming sample extraction process before amplification [5]. In general, RNA isolation from clinical specimens requires extraction, purification, and elution steps, which is a major bottleneck of the PCR method for SARS-CoV-2 detection. At the beginning of the pandemic, the most common commercial extraction methods for RT-PCR analysis were based on silica-column or magnetic-bead extraction methods, but both are laborious and expensive. Recently, many RNA extraction-free methods have been described and unanimously suggest that nucleic acid extraction-free detection of SARS-CoV-2 is feasible [6], and the extraction-free method could be beneficial for mass screening with less effort, time, and cost [7,8]. However, when there is no extraction, there are variations in sensitivity and specificity depending on the RT-qPCR kit used [8], probably because potential inhibitors that are present in the clinical samples can partially or totally inhibit Taq polymerase activity.

An alternative to RT-qPCR are antigen-based lateral flow immunochromatography (ICT) tests that are inexpensive and easy to use, require no equipment, and can be used onsite in mass testing [9]. The main problem of ICT tests is that the sensitivity is five logarithmic orders lower than that of the RT-PCR method [10]. To overcome the drawbacks of RT-qPCR and antigen detection, several isothermal amplification methods have been developed. Among them, the loop-mediated isothermal amplification (LAMP) method is the most used [11]. In a LAMP reaction, the amplification of the nucleic acids occurs at a single temperature; this feature allows for the reaction to be performed in a thermocycler or in simple equipment, such as a heat-block [12], and the results can be seen by several methods like fluorescence [13], colorimetric [14], or electrochemical detection [15,16]. The LAMP technique can be used as a point-of-care detection test or performed in an equipped laboratory in the same way. The time from the sample collection to results is considerably less than that of the PCR test. The LAMP technique has been reported to be more tolerant of inhibitors than RT-PCR and allows better quick sample treatments and even RNA extraction-free procedures [17].

Most of the methods described for the RT-LAMP detection of SARS-CoV-2 are based on commercial colorimetric pH-sensitive detection kits where the pH change of the amplification reaction changes, in turn, the colour of the sample from pink to yellow [14,18,19,20,21]. However, these pH-sensitive RT-LAMP methods are most of the time incompatible with the extraction-free process due to the pH of the sample, especially saliva, and the lack of washing steps, resulting in low sensitivity and false-positive results [22]. To reduce heating steps and avoid centrifugation or dilution in the preparation of the saliva sample, an alternative to the pH-sensitive colorimetric detection is the use of the calcein molecule in the LAMP reaction. This molecule is quenched by a manganese ion. When the amplification occurs, magnesium ions are generated and the calcein molecules bind them, changing their conformation and emitting fluorescence, the colour in the sample changing then from orange to green. This molecule allows double detection of fluorescence and colorimetry [23]. 

In this work, we have compared a new calcein RT-LAMP reaction with several commercial colorimetric and fluorescence-based RT-LAMPs with the aim of proposing an extraction-free colorimetric RT-LAMP for the detection of the SARS-CoV-2 virus in saliva. Combining a heat treatment of the sample and a double colorimetric and fluorescence detection of the LAMP products would be a good solution for mass screenings of the population when new outbreaks occur, and thus relieve the pressure from healthcare services. 

## 2. Materials and Methods

### 2.1. Reference Method (RT-qPCR)

The clinical samples were collected in saliva viral transport medium from Vircell, Granada, Spain. Total nucleic acids of the saliva samples were extracted using the QIAamp Viral RNA mini kit (Qiagen, Hilden, Germany) following the manufacturer’s instructions. Nucleic acids from samples collected at the Microbiology Department at University Hospital Donostia (UHD), Donostia-San Sebastián, Spain, were automatically extracted and purified using magnetic beads (StarMag, Seegene, Seoul, Republic of Korea).

The RT-qPCR method was used as the reference method and to check if the samples were positive or negative. The target region was a 72 bp fragment of the Nucleocapsid (N) gene, and the primers used were recommended by the WHO for SARS-CoV-2 diagnostics [24]. The reaction consisted in 10 µL of One-step PrimeScript III RT-qPCR mix (Takara, Japan) 2× Buffer, 200 nM of the probe (5′-FAM-ACC CCG CAT TAC GTT TGG TGG ACC-BHQ1-3′), 375 nM of forward (5′-GAC CCC AAA ATC AGC GAA AT-3′) and reverse (5′-TCT GGT TAC TGC CAG TTG AAT CTG-3′) primers (Biomers, Ulm, Germany), RNase/DNase-free water, and 5 µL of the sample in a total reaction volume of 20 µL. The amplification reaction was as follows: 53 °C for 5 min, 95 °C for 10 s, and 50 cycles of 95 °C for 5 s and 55 °C for 30 s, acquiring fluorescence in the green filter with a CFX connect thermocycler (Bio-Rad, Hercules, CA, USA). Clinical samples collected at UHD were tested using the Allplex SARS-CoV-2 RT-qPCR assay (Seegene, Seoul, Republic of Korea), and reactions were monitored by the CFX96 (Bio-Rad, Hercules, CA, USA). 

### 2.2. RT-LAMP

#### 2.2.1. Saliva Samples

For the optimization of the method, 26 saliva samples were used (12 positive and 14 negative for SARS-CoV-2 virus). Samples were self-collected in viral transport medium (Vircell, Spain) from volunteers at the Gaiker Technology Center and stored at −80 °C until used. For the comparative assay of the two colorimetric RT-LAMP reactions (IH-C and WS-PR), an additional 22 saliva samples collected at UHD were tested (12 positive and 10 negative). All saliva samples used in the study were tested by RT-qPCR as gold standard, as described in Section 2.1. 

#### 2.2.2. Saliva Samples Processing

For the LAMP experiments, three different saliva sample processing protocols were tested with the WS-F RT-LAMP (see Table 1). The first one consisted in heating the sample at 95 °C for 10 min in a heating block. The second was the same as the first one but making ½ dilution in RNase/DNase-free water (Qiagen, Germany) after heating the sample to minimize possible inhibitions of the reaction. The last protocol used QuickExtract solution (Lucigen, Teddington, UK) as a lysis buffer, in which the samples were mixed 1:1 and heated at 65 °C for 15 min and then at 98 °C for 2 min.

#### 2.2.3. RT-LAMP Mixes, Primer Sets and Measure Principles

In this work, different RT-LAMP premixes were used: manually prepared vs. commercial, colorimetric vs. fluorometric, and newly designed primer sets vs. commercial primers, as shown in Table 1. The commercial premixes were chosen as they are the most commonly used by RT-LAMP users and those previously used in UHD.

The WS-C premix consisted of 12.5 µL of Master Mix, 4 µL of ND3B primers, 1 µL of calcein 625 µM, 1 U µL^−1^ of RiboGuard RNase Inhibitor (Lucigen, UK), and RNase/DNase-free water in a final volume of 25 µL.

#### 2.2.4. In-House RT-LAMP Detection 

The in-house RT-LAMP (IH) was performed using ND3B primers designed with the software PrimerExplorerv5 that specifically target a region of the N gene of the SARS-CoV-2 virus (Table 2), the premix described in Table 1, and fluorescent (IH-F) or colorimetric (IH-C) detection. The performance efficiency of ND3B primers was assessed using different concentrations of MgSO_4_, betaine, primers, and enzyme units combined with the reaction temperature. This study can only be performed with in-house premixes (IH-C/F) because commercial premixes have a fixed combination of the components. Therefore, the IH-C/F were optimized for the newly designed ND3B primers.

The limit of detection (LoD) was established using the commercial Amplirun quantified synthetic RNAs from SARS-CoV-2 (Vircell, Spain). Serial dilutions were done in 10 replicates from 1000 to 16 copies. LoD was established at the concentration at which all the replicates were positive. For the specificity assay, primers were tested with SARS-CoV-2 RNA as the positive control and RNA from OC43 coronavirus, enterovirus 68, MERS coronavirus, Rhinovirus, and 2003 SARS-CoV (Vircell, Spain) quantified at 1000 copies. 

The analytical LoD was determined with the IH-F reaction performed at 63 °C for 60 min, monitored with a real time CFX Connect thermocycler at 60 s intervals, and read with the green filter. The IH-C reaction consisted of the same components used for the LoD study, except for Evagreen, which was replaced with calcein, a dye molecule that is quenched when it binds to the manganese ions in the reaction [25]. The results of the amplification reaction were visualized with both fluorescence and the naked eye.

#### 2.2.5. Limit of Detection in Saliva and Validation of Different RT-LAMP Assays

The LoD in saliva was established using two-fold serial dilutions of a positive saliva sample (10S) in a negative saliva sample (0S) to maintain the same amount of saliva in the reaction. The sample (10S) had been previously quantified by RT-qPCR to estimate the viral load (viral copies). The study of the clinical LoD was performed using the WS-F and the IH-F assays.

All clinical samples were tested with the newly developed dual WS-C reaction completed in a RotorGene thermocycler (Qiagen, Germany) at 63 °C for 60 min, acquiring fluorescence in green filter at 60 s intervals. The colorimetric results were visualized with the naked eye.

For comparison of the calcein and commercial RT-LAMP assays, the 22 hospital clinical samples were tested with the developed WS-C (colorimetric and fluorescent detection) and two commercial RT-LAMP kits, WS-F and WS-PR, for the fluorometric and colorimetric detection, respectively. Colorimetric results were analysed with the naked eye.

## 3. Results and Discussion

### 3.1. Limit of Detection and Specificity of the Newly Designed RT-LAMP Primers

LoD achieved with the IH-F premix, using the newly designed ND3B primers, was monitored in real time by fluorescence and established according to the time to detection (TTD). TTD represents the time in minutes between the start of the reaction and the beginning of the amplification process when the fluorescence emission starts to be detected. Table 3 represents the TTD results versus SARS-CoV-2 RNA copies. The LoD achieved for the ND3B primers set was established in 62 copies.

**Figure 1:**
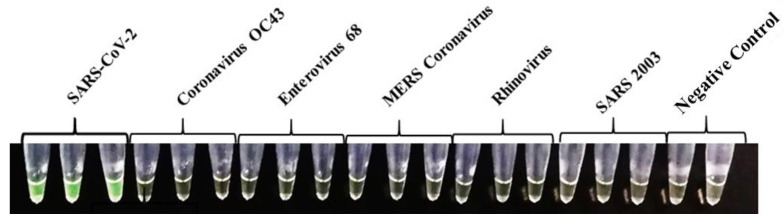
Colorimetric results of the specificity assay against different related coronaviruses and other respiratory viruses obtained with IH-C. Positive SARS-CoV-2 samples are in green and negative samples are in orange.

To assess the specificity of the ND3B primers, the IH-C was performed with commercial RNA from five related coronaviruses and other common respiratory viruses (Vircell, Spain). SARS-CoV-2 RNA was used as positive control. Results were observed at the endpoint by a colour change to green in the positive control and no change for the negative ones. The colorimetric results of the specificity assay performed with the IH-C for the ND3B primers set can be seen in Figure 1. All viruses were tested in triplicates. The reaction was specific for the SARS-CoV-2 virus and no cross-reactivity was observed with the other tested viruses.

### 3.2. Sample Pretreatment Optimization

Numerous studies have shown that saliva is an equally sensitive sample as nasopharyngeal swabs for SARS-CoV-2 detection [26,27,28]. Compared to swab-based methods, saliva collection is minimally invasive and can be reliably self-collected without the need for qualified personnel. Several easy methods of saliva preparation in combination with RT-LAMP, based on simple steps such as heating the sample [29] or adding a buffer lysis before amplification, [17] have been used for SARS-CoV-2 virus detection. For direct detection of SARS-CoV-2 in saliva, Catarina et al. describes a centrifugation step and dilution of the supernatant with TE to buffer basal pH differences [30], and Lalli et al. heated the saliva sample at 65 °C for 15 min, followed by 95 °C for 5 min, and cooled to 4 °C for 5 min. Then, samples were treated with proteinase k and diluted in TE [31].The above techniques require several tedious steps when processing large numbers of samples, such as centrifugation and sample dilutions for pH neutralization. Pre-treatment of samples should be faster and easier to adapt to a method for mass population screening. One-step pre-treatment will save time in getting results and would facilitate decision-making in the emergencies of hospitals and outpatient clinics. For this reason, three different fast sample treatment protocols were tested and compared with the reference extraction protocol based on silica-column. Very rapid degradation of RNA was observed in saliva samples artificially spiked with viral RNA. Therefore, for all three fast protocols, an RNase inhibitor was added in the premix of the RT-LAMP reaction to prevent degradation of the RNA during amplification.

For the comparation of the sample fast treatment methods, 12 saliva samples shown as positive for the SARS-CoV-2 virus by RT-qPCR were used and tested with the WS-F premix and ND3B primers. Samples Ct were between 21 and 37. With the Qiagen silica-column kit used as the extraction reference method, 10 out of 12 positive samples were detected with a sensitivity of 83%. Among the fast methods, the heating method obtained the best results: 10 samples were positive and 2 negative with a sensitivity of 83%, the same as the reference method. The heating plus dilution method and the QuickExtract protocol detected 8 and 9 positive samples, with a sensitivity of 67% and 75%, respectively (Table 4); as these methods imply a dilution of the sample, the sensitivity could be affected. The two samples that were not detected with the four extraction protocols and RT-LAMP corresponded to the saliva samples with the lowest viral load, with a RT-qPCR Ct of 35.03 and 36.44. These two samples are below the limit of detection of the ND3B primers and also below the limit of detection of antigen-tests [10]. Although the reference and heating methods show the same sensitivity, the heating method has an improvement in the time per sample needed to perform the extraction process, from 40 to 10 min. With the results obtained, the heating method was selected as the best pre-treatment for the rest of the study. Another advantage of this extraction-free method is that heating the sample at 95 °C inactivates it [32], allowing its manipulation out of a biosafety cabinet.

### 3.3. Limit of Detection with Clinical Samples

The LoD in clinical samples was established by combining the extraction-free protocol (heating at 95 °C for 10 min) with the RT-LAMP method. For this purpose, two-fold serial dilutions were done with a positive saliva sample by RT-PCR and Ct 30.89 diluted in a negative saliva sample to maintain the same saliva proportion. Each dilution was performed in duplicates. The saliva sample was quantified using a standard curve of three concentration levels of SARS-CoV-2 viral RNA (from 6250 to 62.5 copies) and R^2^ 0.998. The calculated viral load of the sample was 2.2 × 10^5^ copies/mL corresponding to 1114 copies.

LoD was studied in duplicates with the WS-F premix and the IH-F. A total of 557 copies were detected with the WS-F premix corresponding to dilution −1, and one of the two replicates of dilutions −2 and −3. A total of 278 copies were detected with IH-F corresponding to dilution −2 and detecting one of the replicates of dilution −3, improving the LoD obtained with the commercial WS-F premix. This improvement is due to the fact that the components and concentrations of the IH-F premix are adjusted for the ND3B primers set, which facilitates the reaction performance in contrast to the commercial WS-F premix with the concentration of components already set without any possibility of adjustment.

### 3.4. Clinical Validation of the Colorimetric RT-LAMP

For the clinical validation of the extraction-free colorimetric WS-C detection method developed, 26 saliva samples were tested (12 positive and 14 negative). To test the most suitable method for use in healthcare facilities and for mass screening, the commercial premix WarmStart LAMP kit was selected instead of the in-house premix to facilitate the reaction preparation process; moreover, the combination with the calcein colorimetric detection instead of fluorometric detection only facilitates the possibility to perform the assay with a conventional heater without the need for a thermocycler for fluorescence reading.

**Figure 2:**
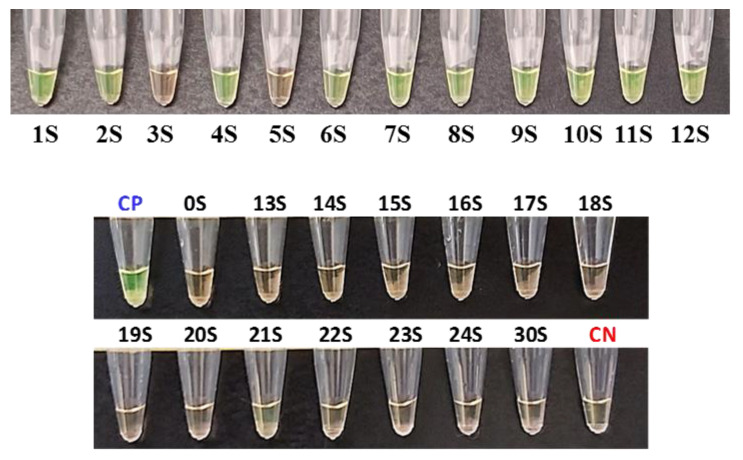
Sensitivity results of the developed WS-C with saliva samples. Positive results in green and negative ones in orange. CP: Positive control. CN: Negative control.

All samples were assayed and quantified by RT-qPCR to have an estimation of the viral load of the positive samples (Table 4). The sensitivity and specificity of the extraction-free WS-C were 83% and 100% (Figure 2). Samples 3S and 5S were detected as negative by the WS-C, whereas in the RT-qPCR they were positive, but with a very low viral load (RT-qPCR Ct of 36.44 and 35.03, respectively).

**Figure 3:**
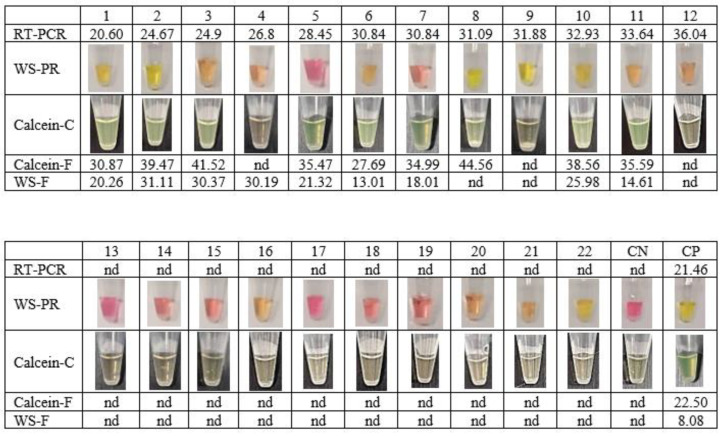
Sensitivity comparation of the developed extraction-free WS-C against a commercial colorimetric RT-LAMP (WS-PR) and commercial fluorescent RT-LAMP (WS-F). CN: Negative control. CP: Positive Control. nd: not detected.

To verify the achievement of the new extraction-free WS-C, a comparative assay was performed using clinical saliva samples processed by RT-qPCR at the hospital. For this purpose, two commercial RT-LAMP based kits were compared to the developed WS-C: WarmStart Colorimetric RT-LAMP kit (WS-PR) and WarmStart Fluorescence LAMP/RT-LAMP kit (WS-F) with NEB primers. WS-C results were analysed simultaneously by fluorescence (Calcein-F) and colour change (Calcein-C). As shown in Figure 3, 12 saliva samples were positive with Ct values between 20 and 36 and 10 saliva samples were negative according to the results obtained with the referenced RT-qPCR method.

The extraction-free method was used prior to all RT-LAMP premix options. Samples 9 and 12 were not detected by any of the RT-LAMP methods. This could be due to the Ct of the samples being close to the LoD, 31.88 and 36.04, respectively. The WS-PR kit obtained 40% specificity compared to the RT-qPCR method (Table 5). These results are probably due to the variability in saliva pH in extraction-free samples. These results confirmed (Figure 3) that the WS-PR colorimetric reaction is not suitable for extraction-free saliva samples. In contrast, the WS-C developed obtained 100% of specificity and sensitivity of 75% compared to the RT-qPCR method. These results are equivalent to those obtained with the WS-F, with the improvement that can be read not only by fluorescence, but also with the naked eye.

Considering all saliva samples tested in this study (Figure 2 and Figure 3), the sensitivity of the developed method reaches 79%, and for samples with Ct ≤ 34, the sensitivity reaches 90%. These results suggest that this could be a valuable technique for SARS-CoV-2 detection in saliva samples, and the method could be used as an alternative to antigen tests in healthcare services.

## 4. Conclusions

In conclusion, a sensitive, specific, and fast technique for the diagnostic of SARS-CoV-2 in saliva samples has been developed. The optimized workflow combines a sample heating step with an extraction-free WS-C amplification. Results can be detected by a simple colour change in the reaction tube that distinguishes between positive and negative results. The WS-C shows a sensitivity of 90% for samples with a Ct ≤ 34 and a specificity of a 100%. The developed method could be used in the future as a mass-screening tool or as an alternative to the antigen test in hospitals and healthcare services.

## Figures and Tables

**Table 1:** Different RT-LAMP mixes used in the study, indicating the type of premix, dye, primers, type of detection, and experiment in which they were used.

Acronym	RT-LAMP	Premix Type	Primers	Dye	Detection	Experiment (Section)
IH-F	In-House RT-LAMP	Manual *	ND3B	Evagreen (25 µM)	Fluorescent	ND3B LoD (Section 3.1) Extraction-free LoD (Section 3.3)
IH-C	In-House Calcein RT-LAMP	Manual	ND3B	Calcein (625 µM)	Colorimetric and Fluorescent	ND3B specificity assay (Section 3.1)
WS-C	WarmStart-Calcein	Commercial **	ND3B	Calcein (625 µM)	Colorimetric and Fluorescent	Clinical validation (Section 3.4) Comparative assay (Section 3.4)
WS-F	WarmStart-F	Commercial	ND3B NEB	Fluorescence dye **	Fluorescent	Pre-Treatment optimization (Section 3.2) Extraction-free clinical LoD (Section 3.3) Comparative assay (Section 3.4)
WS-PR	WarmStart-phenol red	Commercial	NEB	pH-indicator **	Colorimetric	Comparative assay (Section 3.4)

* Manual Premix: 2.5 µL of ThermoPol buffer (10×), 1.5 µL of MgSO_4_ 100 mM, 3.5 µL of dNTP solution mix (New England Biolabs, Hitchin, UK), 4 µL of primers, 2 µL of Betaine (Sigma-Aldrich, Gillingham, UK) 5 M, 1.5 µL of Bst DNA polymerase large fragment 8 U µL^−1^ (New England Biolabs, UK), 0.1 µL of RT Maxima (Fisher Scientific, Waltham, MA, USA) 200 U µL^−1^, and 5 µL of sample in a final volume of 25 µL. ** Commercial: WarmStart LAMP Kit (DNA and RNA, New England Biolabs, UK), WarmStart Fluorescence LAMP/RT-LAMP kit, and WarmStart Colorimetric RT-LAMP kit were used attending to the manufacturer’s instructions.

**Table 2:** Sequence and concentration of the different RT-LAMP primers (Biomers, Germany) used in the study.

Set Name	Primer	Conc. (µM)	Sequence (5′-3′)
ND3B	F3	0.2	CCTTTTACAATTAATTGCCAGGA
	B3	0.2	CCACTGCGTTCTCCATTC
	FIP	1.6	ACACGAACGTCATGATACTCTAAAAACCTAAATTGGGTAGTCTTGT
	BIP	1.6	GGACCCCAAAATCAGCGAAATTGCCAGTTGAATCTGAGG
	LF	0.8	TTCATAGAACGAACAACGCACT
	LB	0.8	GCACCCCGCATTACGTTTG
Gene E1 (NEB) *	F3	0.2	TGAGTACGAACTTATGTACTCAT
	B3	0.2	TTCAGATTTTTAACACGAGAGT
	FIP	1.6	ACCACGAAAGCAAGAAAAAGAAGTTCGTTTCGGAAGAGACAG
	BIP	1.6	TTGCTAGTTACACTAGCCATCCTTAGGTTTTACAAGACTCACGT
	LF	0.4	CGCTATTAACTATTAACG
	LB	0.4	GCGCTTCGATTGTGTGCGT
Gene N2 (NEB)	F3	0.2	ACCAGGAACTAATCAGACAAG
	B3	0.2	GACTTGATCTTTGAAATTTGGATCT
	FIP	1.6	TTCCGAAGAACGCTGAAGCGGAACTGATTACAAACATTGGCC
	BIP	1.6	CGCATTGGCATGGAAGTCACAATTTGATGGCACCTGTGTA
	LF	0.4	GGGGGCAAATTGTGCAATTTG
	LB	0.4	CTTCGGGAACGTGGTTGACC

* NEB: New England Biolabs.

**Table 3:** Limit of detection results of the IH-F reaction performed with the newly designed ND3B set of primers.

RNA Copies	TTD1	TTD2	TTD3	TTD4	TTD5	TTD6	TTD7	TTD8	TTD9	TTD10	X	%
1000	12.5	13.6	13.0	12.3	14.2	12.5	12.9	13.4	12.7	12.3	12.92	100
500	14.7	14.8	14.3	15.1	15.9	13.5	12.2	14.9	13.6	13.0	14.19	100
250	14.0	15.6	16.3	14.6	15.4	14.4	14.4	15.4	18.0	13.2	15.12	100
125	30.5	16.7	13.5	15.9	16.1	30.1	20.8	14.2	27.5	17.5	20.26	100
62	19.6	17.0	17.9	17.2	16.9	18.3	15.2	20.0	14.6	16.7	17.33	100
41	33.2	NEG	18.3	22.5	NEG	20.8	16.4	16.5	18.4	16.3	20.30	80
31	23.0	16.9	NEG	18.6	18.2	15.6	36.3	NEG	21.1	19.0	21.07	80
16	20.2	NEG	34.0	NEG	18.3	NEG	NEG	19.6	NEG	35.3	25.48	50
CN	NEG	NEG	NEG	NEG	NEG	NEG	NEG	NEG	NEG	NEG	-	

TTD: time to detection in minutes. X and %: the mean TTD and the percentage of positive replicates (%). NEG: negative.

**Table 4:** Comparative table of the TTD of the saliva samples with the different extraction protocols.

Sample	QIAGEN Kit	95 °C 10 min	95 °C 10 min Dil. 1/2	QuickExtract	RT-qPCR Ct
1	13.53	16.11	14.88	19.44	21.42
4	14.39	17.3	15.96	16.66	23.35
11	16.36	19.05	17.39	18.37	25.87
2	16.14	28.34	21.69	18.01	27.51
9	16.51	19.35	18.28	19.31	27.76
6	18.06	21.95	Not detected	19.48	28.21
8	17.23	19.43	17.72	18.82	28.37
7	17.19	19.01	15.54	17.73	30.07
10	19.24	22.32	21.63	21.01	30.89
12	18.38	22.09	Not detected	Not detected	31.74
5	Not detected	Not detected	Not detected	Not detected	35.03
3	Not detected	Not detected	Not detected	Not detected	36.44

**Table 5:** Results of the SARS-CoV-2 RT-LAMP methods of 12 positive and 10 negative saliva samples by RT-qPCR.

	True Positive	True Negative	False Negative	False Positive	Sensitivity	Specificity
Calcein-C	9	10	3	-	75%	100%
Calcein-F	9	10	3	-	75%	100%
WS-PR	11	4	1	6	91%	40%
WS-F	9	10	3	-	75%	100%

## Data Availability

The data presented in this study are available on request from the corresponding author.
